# New insights into leishmaniasis in the immunosuppressed

**DOI:** 10.1371/journal.pntd.0006375

**Published:** 2018-05-10

**Authors:** Hannah Akuffo, Carlos Costa, Johan van Griensven, Sakib Burza, Javier Moreno, Mercè Herrero

**Affiliations:** 1 Swedish International Development Agency (Sida), and Microbiology, Tumor and Cell biology (MTC), Karolinska Institutet, Solna, Sweden; 2 Federal University of Piauí, Teresina-PI, Brazil; 3 Department of Clinical Sciences, Institute of Tropical Medicine, Antwerp, Belgium; 4 Médecins Sans Frontières, New Delhi, India; 5 *World Health Centre (WHO) Collaborating Centre for Leishmaniasis*, National Centre for Microbiology, Instituto de Salud Carlos III, Madrid, Spain; 6 Leishmaniasis, IDM Unit, Neglected Tropical Diseases, WHO, Geneva, Switzerland; Pasteur Institute of Iran, ISLAMIC REPUBLIC OF IRAN

## Abstract

Immunosuppression contributes significantly to the caseload of visceral leishmaniasis (VL). HIV coinfection, solid organ transplantation, malnutrition, and helminth infections are the most important immunosuppression-related factors. This review briefly describes the challenges of these associations. East Africa and the Indian subcontinent are the places where HIV imposes the highest burden in VL. In the highlands of Northern Ethiopia, migrant rural workers are at a greater risk of coinfection and malnutrition, while in India, HIV reduces the sustainability of a successful elimination programme. As shown from a longitudinal cohort in Madrid, VL is an additional threat to solid organ transplantation. The association with malnutrition is more complex since it can be both a cause and a consequence of VL. Different regimes for therapy and secondary prevention are discussed as well as the role of nutrients on the prophylaxis of VL in poverty-stricken endemic areas.

## Introduction

Immunosuppression is associated with leishmaniasis. It is more frequently described in association with visceral leishmaniasis (VL) as it is one of the consequences of the disease, especially in the latter stages. VL occurs both in the ‘Old World’ and in the ‘New World’, often in underresourced areas of conflict and instability. The causative agents of VL are those in the *Leishmania donovani*–*L*. *infantum* complex. However, other species of *Leishmania* that cause cutaneous or mucocutaneous leishmaniasis have also been described as resulting in immunosuppression.

Immunosuppression in leishmaniasis may stem from the leishmanial infection itself, but it can be further exacerbated by comorbidities involving coinfections with pathogens, such as Human Immunodeficiency Virus (HIV) and helminths, or through morbidity associated with malnutrition.

## Methods

Treatment of VL is difficult in patients with certain comorbidities. This paper, developed after deliberations at a session on the subject at the WorldLeish 2017, discusses the value of antiretroviral treatment (ART) in VL–HIV patients, illustrates the contrasting picture of VL–HIV in East Africa and Asia, examines VL in immunosuppressed patients after transplantation, and considers the need for nutritional supplements as a treatment adjunct in VL-endemic countries with widespread malnutrition. We elaborate on how immunosuppression due to HIV coinfection and malnutrition exacerbates the symptoms of VL as well complicates treatment. We illustrate the many things that we do not understand to date but also other things where there remains a ‘know–do gap’, a gap between evidence and effective implementation.

## Results

### Helminths

Experimental coinfection with *Schistosoma mansoni* and *L*. *donovani* in mice showed that those with established *S*. *mansoni* infections fail to control *L*. *donovani* growth in the liver and spleen [1]. The influence of helminth coinfection on cutaneous leishmaniasis has also been presented in a number of studies but with conflicting results, which may be because the impact of helminth coinfection on leishmanial lesion growth appears to be time dependent [2].

Furthermore, helminth infections have been reported to influence the clinical course and the immune response to cutaneous leishmaniasis caused by *L*. *braziliensis*. Patients with *L*. *braziliensis* coinfected with helminths healed at a slower rate compared to patients without coinfection, suggesting a role for screening and treatment of helminths to improve the outcomes of *L*. *braziliensis* treatment and potentially reduce the risk of progression to mucosal disease [3]. However, subsequent randomised clinical trials showed that treating helminths did not make a difference in treatment outcomes [4].

### HIV

HIV infection has been shown to appreciably increase the risk of developing VL in endemic areas, reduce the chances for adequate therapeutic response, and greatly increase the likelihood of relapse. In vitro studies showed that the addition of HIV to human mononuclear cell cultures altered the T helper cell cytokines induced in response to *L*. *donovani* stimulation [5].

Subsequent studies in Ethiopian HIV-coinfected VL patients demonstrated that the outcome of both conditions was worsened, with enhanced severity of the VL and acceleration of HIV progression.

The current picture of VL seen in East Africa and that in Asia differ considerably, with the picture in India, Nepal, and Bangladesh moving towards elimination of VL as a public health problem based on political commitment coupled with focused studies aimed at improving treatment. The measures taken in this elimination agenda has led to improved surveillance and, in turn, better identification of VL–HIV.

### VL–HIV coinfection in East Africa

East Africa has an estimated VL caseload of around 30,000 cases annually. Several areas face high rates of HIV coinfection. This is most marked in Northwestern Ethiopia, where coinfection rates of approximately 20% have been reported [6]. In part, this relates to the high number of young seasonal workers migrating to the area from VL-nonendemic highlands [6].

As in the rest of the world, the standard of care for VL–HIV coinfected patients in East Africa consists of several components. Besides aiming to achieve parasitologically confirmed VL cure, early initiation of ART is vital. This should be followed by secondary prophylaxis, targeting those at highest risk of VL relapse [6].

Within East Africa, the first-line treatment in HIV-negative patients consists of parenteral administration of antimonials and paromomycin for 17 days. Overall, this combination therapy was found effective and safe, although the daily intramuscular injections can be painful [6]. For HIV patients, WHO guidelines recommend liposomal amphotericin B at a total dose of 40 mg/kg [7]. Although there are a number of commercially available preparations of lipidic and liposomal formulations of amphotericin B available, at present, the only liposomal preparation procured for use in VL by WHO is the liposomal formulation AmBisome (Gilead Sciences; San Dimas, California, United States of America). There are a number of noninferiority studies ongoing with other preparations; however, to date, safety and effectiveness results have not been encouraging [8]. Additionally, the lack of a clear regulatory pathway for the registration of these other formulations complicates the entry of new competitors on the markets [8]. As such, outside of nongovernmental organisation (NGO) settings, availability is often limited.

Regarding treatment of VL in HIV-positive patients, most drugs have proven disappointing (Table 1). The Médecins Sans Frontières (MSF) experience with a combination regimen consisting of AmBisome (Gilead Sciences; San Dimas, California, USA) 30 mg/kg and miltefosine for 28 days looks promising, with initial cure rates of 81% [6]. Based on this experience, Drugs for Neglected Diseases *initiative* (DND*i*) is conducting a randomised controlled trial with two arms: (1) AmBisome 30 mg/kg combined with miltefosine for 28 days and (2) AmBisome 40 mg/kg in monotherapy [9]. Data are expected to be reported soon.

**Table 1:** Overview of current evidence related to different aspects of VL–HIV care in East Africa.

Objective	Status: Experience in Northwestern Ethiopia
Achieving parasitological cure	Antimonials: toxicity, suboptimal efficacy [10] Miltefosine: safe but limited efficacy [11] AmBisome 30 mg/kg: safe but limited efficacy [12] • Initial treatment cure rate of 74% in primary VL and 38% in relapsed VL; overall cure rate 59% AmBisome 30 mg/kg IV + miltefosine PO for 28 days in compassionate use [6] • Initial cure rate of 81%
Early ART initiation	Retrospective patient file review in one referral and one district hospital in Northwestern Ethiopia. Amongst newly diagnosed VL–HIV patients, ART uptake was 28% (13/47) at the district hospital and 61% (30/49) at the referral hospital [13]
Preventing VL relapse (secondary prophylaxis)	Single-arm clinical trial evaluating monthly administration of pentamidine 4 mg/kg IV for a minimum of 12 months; a 6-month extension was given for those with CD4 counts ≤ 200 cells/μL by 12 months of pentamidine [6, 14]. No relapse after pentamidine discontinuation if CD4 counts > 200 cells/μL by 12 months of pentamidine (0/28) 3 out of 17 relapses in those with CD4 counts ≤ 200 cells/μL by 12 months, despite a 6-month pentamidine extension
Preventing primary VL (primary prophylaxis)	PreLeisH study (Northern Ethiopia): Multicentre observational cohort study HIV patients in HIV care and living in a VL-endemic area will be followed for 2 years with clinical/laboratory evaluation every 3 months. The incidence of asymptomatic *Leishmania* infection will be determined, and a clinical prediction tool to predict the onset of VL will be developed [15].

**Abbreviations:** ART, antiretroviral treatment; CD4, cluster of differentiation 4; IV, intravenously; PO, per os (oral treatment); VL, visceral leishmaniasis.

Several studies from Ethiopia have shown the importance of ART initiation in VL–HIV coinfected patients to reduce mortality and relapse rates [6, 16]. However, studies looking at ART uptake in routine programme conditions are lacking. In a retrospective patient file review conducted in Northern Ethiopia, ART uptake—defined as documented proof of ART prescription in VL patients—in newly diagnosed VL–HIV patients was fair (61%) at the referral-hospital level but poor (28%) at the district hospital [13] (Table 1). This poor uptake could be due to many factors, including poor documentation and information exchange between referring and recipient facilities. Another factor is that VL–HIV coinfection predominantly occurs in highly mobile populations (e.g., seasonal migrants), who might move back to their place of origin for ART initiation. Alternatively, it is possible that a substantial proportion become lost to follow-up and never start ART, or with long delays.

Data on secondary prophylaxis of VL in HIV patients in anthroponotic (*L*. *donovani*) areas are limited to a single-arm trial, evaluating the use of monthly administration of pentamidine, a drug currently not in use for VL treatment in East Africa. Previously, the outcomes at 12 months of pentamidine use supporting the effectiveness, safety, and feasibility of this intervention have been reported [17]. Data on the risk of relapse after discontinuing 12 months of pentamidine have been published recently [6, 14]. For those reaching a cluster of differentiation 4 (CD4) count > 200 cells/μL at 12 months, no relapses were seen (Table 1). Those with a CD4 count ≤ 200 cells/μL remained at risk, suggesting that pentamidine continuation might be needed on a case-by-case basis until CD4 count levels have increased. The overall risk of VL relapse was 37% by 2 years after starting pentamidine, highest amongst those with low baseline CD4 counts and a history of (multiple) relapses. The Ethiopian national programme should therefore consider this intervention within their national guidelines.

An unexplored strategy to reduce the VL–HIV burden would entail the prevention of VL onset in HIV patients. The PreLeisH study, started in October 2017, aims to provide insights into the incidence of asymptomatic *Leishmania* infection in HIV patients living in VL-endemic areas and the identification of those at highest risk of VL [15]. This would constitute a first step towards a ‘screen and treat’ strategy [18].

### VL–HIV coinfection in Asia

The incidence of VL has been decreasing in Asia over the last decade. India, Nepal, and Bangladesh used to carry over 50% of the global burden of VL; however, reported cases of VL are now substantially higher in East Africa than in the Indian subcontinent (ISC) [19]. The decline in cases is most likely due to the elimination efforts because in 2005, the three most affected countries entered into an agreement to eliminate VL as ‘a public health problem’ by 2015, which was later extended to 2017. This was considered feasible due to a myriad of factors, including the assumed purely anthroponotic nature of VL spread in the subcontinent, a single vector, availability of effective rapid diagnostic tests, and crucially, the availability of short-course effective treatments [20].

The East Indian state of Bihar has been the epicentre of VL for over a century. This populous state is also one of the few in India where the rate of new HIV infections is increasing [21], bringing together two diseases whose interaction is a well-established risk factor for poor outcomes [22]. However, until recently, there has been a dearth of data and evidence surrounding VL–HIV coinfection in the ISC. A 2014 single-centre study from Bihar reported 5.6% of 2,077 consecutive confirmed VL patients ≥14 years of age were found to be HIV positive; half of these were unaware of their HIV status [23]. With improved disease surveillance and reporting, including recommendations to offer all VL patients screening for HIV [20], the numbers of reported coinfected cases are increasing. With the overall falling incidence of VL, this means that at a state level, currently up to 7% of all reported VL patients ≥18 years of age are coinfected with HIV in 2017; however, in highly VL-endemic districts with reliable HIV screening, this is as high as 20% [24].

More recently, increasing numbers of coinfected cases are being reported following the introduction of routine HIV screening in Nepal, where 9 out of 48 VL cases presenting to a tertiary hospital catering to a wide VL-endemic area during 2016–2017 were coinfected with HIV [24]. Bangladesh remains a lacuna since HIV screening of VL patients has not yet been introduced, even in patients presenting with multiple relapses.

There remains a substantial number of issues in the diagnosis and management of coinfected patients in the ISC. Although the recombinant K39 (rK39) test has been shown to be highly sensitive and specific for patients not diagnosed with HIV, its accuracy in coinfected patients has not yet been established in the ISC. Additionally, no high-quality evidence-based treatment regimens exist for coinfection in the ISC; the current WHO and regional recommendation of 40 mg/kg of AmBisome over 38 days is based on evidence generated on a different strain (*L*. *infantum*) in southern Europe (Table 2). A clinical trial to establish the safety and effectiveness of this regimen and that of a shorter lower-dose combination of AmBisome and miltefosine in Bihar is nearing completion [25]. The main challenges for coinfection in the ISC are presented in Table 3.

**Table 2:** Current evidence for treatment of VL–HIV coinfection in the ISC.

Treatment of primary VL–HIV episode	Result	Limitations/Observations
40 mg/kg of liposomal amphotericin B in 10 divided doses on days 1–5, 10, 17, 24, 31, and 38	8 out of 10 relapsed within 7 months, 2 out of 10 defaulted. No patients on ART	Prospective cohort study of 10 patients in southern Europe, pre-ART [26]. No data from ISC available
30 mg/kg AmBisome in 6 divided doses with 100 mg per day oral miltefosine over 14 days	In patients taking ART, 6.4% relapse, 11.2% mortality at 12 months	Observational data from Bihar, India [27]
20–25 mg/kg AmBisome in 4–5 divided doses over 4–5 days	In patients taking ART, 16.2% relapse, 8.7% mortality at 12 months	Observational data from Bihar, India [28]
Treatment of relapses or refractory cases		
No evidence base currently exists		
Secondary prophylaxis		
1 mg/kg amphotericin B deoxycholate or liposomal amphotericin B	No relapse versus 75% relapse in nonprophylaxis arm at 6 months	Retrospective study from West Bengal, India [29].

**Abbreviations:** ART, antiretroviral therapy; ISC, Indian subcontinent; VL, visceral leishmaniasis.

**Table 3:** Challenges in VL–HIV infection in Asia.

Epidemiological
Limited evidence on prevalence of HIV in reported VL cases in endemic areas
No established method of screening HIV patients in VL-endemic areas for VL
No data on prevalence of asymptomatic VL infection in HIV patients in endemic areas
No data on risk factors for progression from asymptomatic VL infection to symptomatic VL infection in patients with HIV
Diagnostic
No evidence on the accuracy of existing RDTs for VL in coinfected patients
Standard VL case definition unlikely to be appropriate for VL–HIV coinfected cases
Improved biomarkers for confirming relapse need to be developed to reduce risk of repeated invasive biopsy
Treatment
No evidence for 40 mg/kg WHO recommended dose for VL–HIV infection in ISC
Only observational data for lower doses of AmBisome and combination treatment
No evidence on primary prophylaxis
Limited evidence on secondary prophylaxis
Very limited therapeutic options for treatment of VL in coinfection
Challenge of VL–HIV–TB triple infection emerging and poorly understood

**Abbreviations:** ISC, Indian subcontinent; RDT, rapid diagnostic test; TB, tuberculosis; VL, visceral leishmaniasis.

Late presentation of coinfected patients poses the most difficult obstacle in improving patient outcomes. Nearly half present with CD4 counts of under 100, with the majority under 200 [28, 30]. Concomitant infection with TB has become an additional challenge; with the use of cartridge-based nucleic amplification test (CB-NAAT) screening in VL–HIV coinfected patients, up to 20% are being identified with tuberculosis (TB) infection. These patients have the highest risk for mortality [28] and present therapeutic challenges with regards to timing and monitoring of concurrent treatment initiation (or continuation) for VL, TB, and HIV. As such, in endemic areas, VL should always be considered in addition to other opportunistic infections already highlighted by recent WHO guidelines on management of patients presenting with advanced HIV disease [27].

In the future, a better understanding of the epidemiology and progression of VL in patients with HIV through improved proxy biomarkers will be key in improving earlier detection and outcomes. The susceptibility of *L*. *donovani* in the ISC to low-dose liposomal amphotericin B may provide a unique opportunity for primary prophylaxis in asymptomatic VL–HIV coinfected patients, potentially reducing progression to symptomatic disease, and should be further explored.

### Solid organ transplants (SOTs) and leishmaniasis

The worldwide number of cases of VL in recipients of SOTs has quadrupled since the 1990s, although VL is still a rare disease among transplant recipients [29]. Most of the clinical cases have been reported in the Mediterranean Basin, particularly in Spain, which is among the foremost in performing SOTs and is endemic for *L*. *infantum* transmission. In these patients, the immunosuppressive therapy to avoid graft rejection affects T cell lymphocytes, altering the mechanisms of defence against intracellular microorganisms as *Leishmania*, predisposing to manifestations of disease. However, the precise mechanism behind the VL risk has not been well defined.

The development of VL by SOT recipients is not related to the origin of the organ, the socioeconomic category of the patient, nor the type of transplant, although usually associated with renal transplants as they are the most frequent transplant (Table 4). It is much more dependent on the degree of exposure to infection. The higher incidence of VL in SOT recipients from Brazil compared to those from Spain has been explained as a consequence of the higher incidence and transmission of VL in the first place [31].

**Table 4:** Review of publications reporting cases of leishmaniasis in SOT recipients, describing the organ transplanted and the number of cases and the treatment options used for each type of leishmaniasis.

Type of leishmaniasis	Type of SOT	Cases reported	References	Treatments
Visceral leishmaniasis	Kidney	119	[31, 33, 38–58]	Primary: AmBisome, Antimonials, Amphotericin B, Allopurinol Relapses: AmBisome, Miltefosine
	Liver	11	[31, 56–60]	
	Heart	9	[31, 33, 58, 61, 62]	
	Lung	4	[31, 63, 64]	
	Pancreas	2	[65, 66]	
Visceral and cutaneous	Kidney	2	[67, 68]	AmBisome B, Antimonials
	Liver	1	[69]	
Cutaneous leishmaniasis	Kidney	5	[70–74]	Antimonials, Ambisome B, Allopurinol + fluconazole
Mucocutaneous leishmaniasis	Kidney	3	[75–77]	Antimonials
	Liver	2	[78, 79]	Amphotericin B, AmBisome
Asymptomatic leishmaniasis	Kidney	42	[35, 80]	
	Liver	4	[81]	

**Abbreviation:** SOT, solid organ transplant.

The recent outbreak of leishmaniasis in Fuenlabrada (southwest Madrid, Spain), with a mean incidence rate of 22.2 per 100,000 inhabitants in the general population [32], has allowed the study of a cohort of patients with SOT living in the area [33]. During the outbreak, a total of 7 VL cases were counted amongst the 68 SOT recipients included in the study, yielding an annual incidence of 2,997 cases per 100,000 population. This suggests that the susceptibility to develop VL is 135 times higher in these patients than shown by the immunocompetent individuals living in the same area. This study also confirms that the degree of exposure to infection, expressed as the distance from the patient’s residence to the focus of the outbreak, is a key risk factor, as has already been described for the general population [34]. Further, despite this higher risk of developing clinical leishmaniasis, *Leishmania*-specific cellular immunity analysis of the SOT-recipient cohort demonstrated asymptomatic infection in 12 out of the 57 evaluated, all of whom showed specific lymphoproliferation and interferon gamma (IFN-γ) production in response to the parasite antigens. Although the ratio clinical/subclinical appears to be high, parasite infection does not always lead to overt disease in these transplanted patients [35].

In SOT recipients, performance of diagnostic tests for VL is variable, and a combined approach of parasitological and molecular methods is recommended [36] since serological tests have shown a lower sensitivity [31].

The recommended therapy for VL in transplanted patients is the same as that for immunocompetent individuals: a total dose of 21 mg/kg of AmBisome; however, other therapies, such as antimonial, amphotericin b, or allopurinol, have also been used with varying success (Table 4). Relapse rates in these patients are low and can be resolved with AmBisome or miltefosine treatment (Table 4). Secondary prophylaxis is not usually required. The measurement of the cell-mediated immune response in SOT recipients may help to confirm recovery following VL treatment and to assess the risk of relapse and necessity of secondary prophylaxis. Analysis of *Leishmania*-specific cell immunity in 5 SOT recipients patients that remained relapse-free after VL treatment showed positive lymphoproliferation and IFN-γ production after in vitro cell stimulation [35], in a similar way to that reported for HIV-positive patients after VL treatment [37].

All these data highlight the need to assess previous exposure to the parasite in patients subjected to induced immunosuppression, to counsel recipients in endemic areas to reduce the risk of being bitten by sand flies, and to follow up SOT patients in areas with outbreaks of leishmaniasis.

### Malnutrition and leishmaniasis

VL mostly affects poverty-stricken and often malnourished populations. Data from 29,570 VL patients divided over Brazil, East Africa, Nepal, and India showed that severe malnutrition in VL is frequent in South Asia and East Africa, while it is uncommon in Brazil [82]. The amastigote form of the *Leishmania* parasite attacks the reticuloendothelial system of naive people and causes an infection that either spontaneously resolves (in 90% of patients) or progresses over weeks to months to clinical VL and death, if untreated. Progression to disease depends on the condition of the host; genetic factors, immune suppression, malnutrition, and the presence of other infectious diseases all play a role [83, 84]. Observational studies have suggested that both protein malnutrition and micronutrient deficiencies may speed the progression of leishmaniasis infection [85, 86] and that VL itself worsens malnutrition [87, 88].

#### Protein malnutrition and VL

In a murine model of moderate childhood malnutrition, poly-nutrient deficiency led to a 4–5-fold increase in early visceralisation of *L*. *donovani* following infection and a 16-fold decrease in lymph node barrier function [89], while in a recent study in hamsters infected with *L*. *infantum*, well-nourished hamsters had stronger specific immune responses and lower parasite loads than their malnourished counterparts [90]. Hence, it is likely that malnourished people will have a greatly increased risk of developing VL. Indeed, in a study in Brazil, the parasite burden in children with severe and moderate malnutrition was almost three times higher than in nonmalnourished children [91].

#### Micronutrients and VL

Deficiencies of micronutrients such as iron, iodine, zinc, and vitamin A are known as ‘hidden hunger’ and in developing countries often go hand in hand with acute malnutrition. The poverty-stricken populations affected by VL habitually eat large amounts of staple food crops (maize, wheat, rice) high in calories but lacking sufficient micronutrients, leaving them susceptible to disease. In a study aiming to understand the link between micronutrient deficiencies and VL in Bangladesh, it was found that in a population with poor nutritional status, retinol and zinc levels were lower and C-reactive protein levels higher in patients who developed VL compared to those who remained symptomless after infection [92].

#### The reality in the field: Nutritional support for VL patients

Supportive treatment, like nutritional supplementation, before the start of therapy of VL is recommended both by WHO [7] and in national VL guidelines in most endemic countries. Ready-to-use therapeutic food (RUTF) is available in most hospitals in VL-endemic regions as part of well-financed programmes for the control of TB and HIV. But since VL remains a neglected disease, in practice, VL patients do not receive any nutritional therapy, unless they are less than five years of age and included in UNICEF programmes, coinfected with HIV and/or TB, or specifically supported by NGOs.

Populations affected by VL frequently suffer from malnutrition, including micronutrient deficiencies. Clinical experience suggests that VL patients recover more quickly with nutritional supplementation. There is a need of advocacy to governments and donors to understand the importance of nutrition in VL and include nutritional supplements as part of VL treatment. In Africa, RUTF, which is currently only used in TB and HIV programmes, needs to be available for VL patients as well. There is a need for policy change; WHO and partners should advocate for free nutritional support for VL patients to other agencies such as UNICEF or the World Food Programme (WFP).

Documenting malnutrition in VL is crucial for measuring the problem and acting, but VL control programmes often do not collect any anthropometric data. WHO has recently included anthropometric indicators as part of the global VL surveillance system and recommends countries to collect data on weight and height of all VL patients.

In a recent Cochrane protocol review [93] assessing the effects of oral nutritional supplements in people being treated with antileishmanial drug therapy for VL, the authors found no completed or ongoing studies. This absence of evidence should not be interpreted as the evidence of no effect for nutritional supplements in people with VL. It means that eligible research for this review has not been identified.

## Discussion

Special attention should be paid to immunosuppression among persons at risk of exposure to the viscerotropic species of *Leishmania* in the ISC and East Africa and *L*. *infantum* scattered in Central Asia, the Middle East, Mediterranean Basin, and Latin America.

HIV-infected individuals, SOT patients (and, by extension, those taking immunosuppressive medications for other reasons), and malnourished populations are the most vulnerable. They may have a much higher risk of developing disease after being infected as compared to immunocompetent individuals.

Given the reliance on rK39 rapid diagnostic tests (RDTs) in many settings and given the poorer performance of serological testing in immunosuppressed patients, alternative noninvasive diagnostic methods, such as molecular testing or antigen detection, should be evaluated in immunosuppressed patients and adapted for field use. Such tests might also be better suited to monitor treatment response and to diagnose relapses.

Due to the clearer relationship and higher number of affected patients, HIV infection is the most critical threat. Migrant crop workers in Ethiopia pose a challenge due the temporal work that raises hurdles to HIV adherence and secondary prophylaxis for VL. Despite the rewarding efforts to control the transmission of VL in the ISC, there remains a substantial number of coinfected patients. Since patients with CD4 counts under 200 cells/mm^3^ appear to be at a higher risk of relapse, earlier diagnosis of HIV infection, specific biomarkers of relapse, and the development of improved treatment regimens and options for secondary prophylaxis are urgently needed. In Africa, monthly pentamidine and elsewhere weekly or biweekly liposomal amphotericin B, potentially combined with miltefosine, are the most promising regimes.

The VL epidemic in the urban settlement in Fuenlabrada, Madrid, showed clearly that VL can become an urban disease and poses an extremely high risk of disease among immunocompromised individuals living in the proximity of VL foci. Testing for exposure to *Leishmania*, preventing sand fly bites, and careful follow-up are measures that can reduce the harm of VL in solid transplant patients.

The effect of immunosupression caused by malnutrition needs to be studied more carefully, since most severely or moderately malnourished patients live in the most VL-affected, poverty-stricken regions. Malnutrition appears to be simultaneously a risk for the development of VL after infection and a consequence of the disease, possibly as part of the acute phase reaction of VL. Addressing nutrition in populations not covered by nutritional supplementation that are at risk of VL should be implemented. Studies on the specific components of nutrition associated with the risk of VL development, as well as their role as adjuvant therapy for patients, are needed.

This review has shown that the main impact of immunosuppression in persons exposed to *Leishmania* is increased risk of development of disease after exposure, higher risk of death, and relapse. While it is highly likely that *Leishmania* infection leads to parasite persistence due to the absence of sterilising immunity and that T-cell immune response impairment is the principal mechanism behind evolution of disease, the detailed mechanisms of immunosuppression and why some persons are at higher risk than others remain unclear. Moreover, the absence of effective human VL vaccines remains a critical gap in preventing disease and improving outcomes for this group of patients.

The lack of curative, sterilising therapy points the solution on the development of new, more effective, and less toxic oral drugs coupled with the finding of prognostic biomarkers for early detection of relapse. The development of safe immunotherapy, as recently proposed, seems to be an additional option [94]. Finally, the stabilisation and even increase of HIV transmission in many parts of the world, the advancements in organ transplantation techniques, the persistence of hunger and malnutrition in a scenario of globalisation and global warming, and the expansion and reemergence of VL in parts of the world like South America [95], Madrid [32], and Tbilisi [96] raise red flags and stress the priority to be given to this growing global health challenge.

**Key learning points**

- Migrant rural workers in Ethiopia are at high risk of coinfection with HIV, malnutrition, and VL.
- In the ISC, there are limited data on the prevalence of coinfection outside of India, making it a threat for sustained elimination of VL.
- VL is a threat to SOT in endemic areas.
- Nutrient supplementation can help to prevent and to treat VL in endemic poverty-stricken areas.

**Top five papers**

1. Alvar J, Aparicio P, Aseffa A, Den Boer M, Canavate C, Dedet JP, et al. The relationship between leishmaniasis and AIDS: the second 10 years. Clin Microbiol Rev. 2008;21(2):334–59.
2. Diro E, Ritmeijer K, Boelaert M, Alves F, Mohammed R, Abongomera C, Ravinetto R, De Crop M, Fikre H, Adera C, Colebunders R, van Loen H, Menten J, Lynen L, Hailu A, van Griensven J. Use of pentamidine as secondary prophylaxis to prevent Visceral Leishmaniasis relapse in HIV infected patients, the first twelve months of a prospective cohort study. PLoS Negl Trop Dis. 2015;9(10):e0004087.
3. Burza S, Mahajan R, Sanz MG, Sunyoto T, Kumar R, Mitra G, et al. HIV and visceral leishmaniasis coinfection in Bihar, India: an underrecognized and underdiagnosed threat against elimination. Clin Infect Dis. 2014;59(4):552–555.
4. Clemente W, Vidal E, Girão E, Ramos AS, Govedic F, Merino E, Muñoz P et al. Risk factors, clinical features and outcomes of visceral leishmaniasis in solid-organ transplant recipients: a retrospective multicenter case-control study. Clin Microbiol Infect. 2015;21(1):89–95.
5. Badaró R, Jones TC, Lorenço R, Cerf BJ, Sampaio D, Carvalho EM et al. A prospective study of visceral leishmaniasis in an endemic area of Brazil. J Infect Dis. 1986;154(4):639–49.
